# The Patient Concerns Inventory in head and neck oncology: a structured review of its development, validation and clinical implications

**DOI:** 10.1007/s00405-022-07499-0

**Published:** 2022-07-17

**Authors:** Anastasios Kanatas, Derek Lowe, Simon N. Rogers

**Affiliations:** 1 Leeds Teaching Hospitals and St James Institute of Oncology, Leeds Dental Institute and Leeds General Infirmary, Leeds, UK; 2 Astraglobe Ltd, Congleton, Cheshire UK; 3 Faculty of Health, Social Care and Medicine, Edge Hill University, Ormskirk, L39 4QP UK; 4 Liverpool Head and Neck Centre, Liverpool University Hospital Aintree, Liverpool, UK

**Keywords:** Health Related Quality of life, Patient Concerns Inventory, Head and neck cancer

## Abstract

**Introduction:**

The Patient Concerns Inventory (PCI) is a condition specific prompt list that was initially developed for head and neck cancer (HNC) and is referred to as the PCI–HN. There have been numerous publications regarding the PCI–HN, since it was first published in 2009. To date, there has not been a review of its development, validation and clinical implications. A collation of relevant papers into key sections allows multidisciplinary teams and researchers to have an overview of the PCI–HN’s background, evaluation and utility. This is essential if colleagues are to have confidence in the tool and be able to reflect on how to optimise its use in clinical practice.

**Methods:**

Five search engines were used: EMBASE, Medline, PubMed, CINAHL and Handle-on-QOL for the specific term ‘Patient Concerns Inventory’ up to and including 1st February 2022. In addition, an accumulation of PCI–HN data of 507 HNC patients was drawn from previous studies in Liverpool and Leeds between 2007 and 2020 and was analysed specifically for this paper.

**Results:**

54 papers relating to the PCI–HN were identified. The review is structured into eight sections: (1) What is the PCI–HN and how does it work; (2) Feasibility and acceptability; (3) Psychometrics; (4) Items selected and frequency (5) Associations with Health-Related Quality of Life (HRQOL) and casemix; (6) Other observational studies; (7) Randomised trial evaluation; (8) General discussion and further research.

**Conclusions:**

As the term PCI is quite ubiquitous and produces many hits when searching the literature, this review provides a very concise and convenient historical context for the PCI–HN and collates the current literature.

## Introduction

The treatment for head and neck cancer (HNC) can have a detrimental effect on appearance, speech and swallowing, emotional well-being and social integration [1]. The HNC follow-up clinic is an important opportunity for checking for cancer but also provides an opportunity for clinicians to assess the outcome of treatment and for patients to address issues of concern [2]. In this clinic, time is spent in doctor–patient discussion, and also to complete a physical examination for surveillance. This includes the oral cavity, oropharynx, salivary glands, the cervical regional lymph nodes, and specialised procedures, such as nasendoscopy. It can be difficult to identify those patients who, for whatever reason, suffer in silence [3]. The importance of Health-Related-Quality-of-life (HRQOL) is parallel to survival, but HRQOL measures are limited by their interpretation, domains and scoring. The current unmet needs measures are not specifically validated for HNC patients [4]. A recent systematic review recommended the Patient Concerns Inventory (PCI–HN) for use in the HNC setting to assess unmet needs, based on its focus on HNC and its conceptual coverage [4]. The PCI–HN was developed as a tool to improve patient experience and HRQOL outcome, by allowing patients greater control of their health concerns and needs [5]. The purpose of the PCI–HN is to direct the consultation, help elicit patient concerns and act as a trigger when necessary, for onward referral to other members of the multi-disciplinary team [5]. Before a health status measurement instrument can be used in research or clinical practice, its reliability, validity and responsiveness, should be assessed and considered adequate [6]. Since its publication, the PCI–HN has undergone validation from national and international teams; however, it can be difficult for clinicians, members of the multi-professional team and for researchers to get an overview of the tool’s basis, validation and utility. This is essential if colleagues are to have confidence in the tool and an overview allows reflection on how to optimise its use in clinical practice and research. The aim of this work is to systematically collate all the PCI–HN published research and draw conclusions regarding the measurement properties of the instrument and its potential integration as a standard of care in head and neck oncology.

## Methods

### Search strategy

Five search engines were utilized—EMBASE, PubMed, Medline, CINAHL and HaNDLE-On-QOL. Searches were assisted by Leeds Teaching Hospitals NHS Trust and Liverpool University Hospitals NHS Foundation Trust in September 2021. The search terms were ‘Patient Concerns Inventory’ and ‘questionnaire’; however, these terms were expanded to achieve the most thorough results possible:

1. “Head and cancer” OR “Head and neck carcinoma’’
2. “questionnaire” OR “patient-reported outcome”

**Fig. 1:**
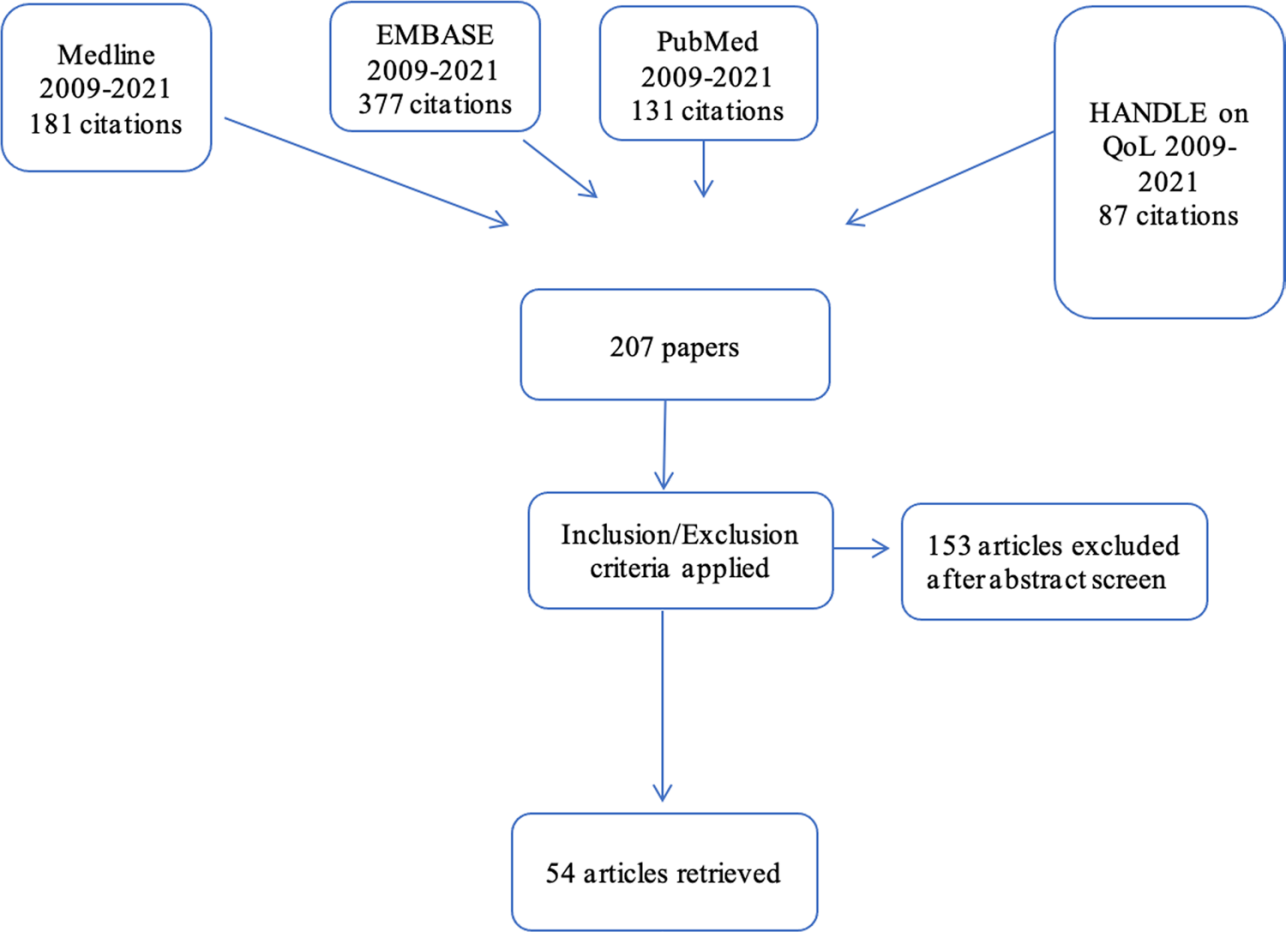
the PRISMA flowchart for this selection process

Preferred reporting items for systematic reviews and meta-analyses guidelines were followed for this systematic review [7]. Figure 1 demonstrates the PRISMA flowchart for this selection process. Quality appraisal and assessment of risk of bias was performed on all included articles by a single author (AK). Quality appraisal was guided by the Joanna Briggs Institute (JBI) critical appraisal checklists [8, 9]. The details of the papers and a summary of conclusions are included in Table 1.

### Selection criteria

We looked at papers from 2009 to 2022 inclusively involving humans of any age, where full text was available in English, including those with non-validated, study-specific questionnaires. The research team included all authors. Results of the literature search were downloaded into an Excel spreadsheet and screened by all three authors (SNR DL, AK) who separately analysed search results. Each paper was categorised by year of publication, title, author(s), cohort, design of study, theme and type of pathology, and then documented as “included, excluded, or unable to decide” from the abstract/title information.

### New analyses

An accumulation of PCI data for 507 HNC patients was drawn from previous studies in Liverpool and Leeds between 2007 and 2020 and was analysed specifically for this paper. Core clinical data (age, gender, primary tumour site, stage and treatment, months from primary diagnosis to PCI–HN clinic) was required and some patients had several PCI–HN records. For this paper the closest available PCI–HN record to 24 months after diagnosis was selected, so long as it was at least 9 months after, median (IQR) 23 (17–41) months. The Mann–Whitney test (2 groups) or Kruskal–Wallis test (3 or more groups) was used to test for differences in the number of PCI–HN items by gender, age at diagnosis group, primary tumour site, stage and treatment. Spearman’s correlation coefficient was used to measure the strength of correlation of the number of PCI–HN items with the six-point overall QOL question scale of the University of Washington QOL questionnaire (UW–QOL) and with the UW–QOL social–emotional and physical function subscale scores.

### Ethical approval

The data were collected as part of the clinical audit process and this part of data was approved by Liverpool University Hospital NHS Foundation Trust Audit Department (CAMS reference number 9939).

## Results

Following the removal of duplicates 207 articles were identified, of which 153 were excluded (not related to head and neck cancer). 54 papers were included in this study.

### What is the PCI–HN and how does it work?

The PCI–HN was first published in 2009 [5] and is a condition-specific prompt list allowing patients to raise concerns that otherwise might be missed [10]. The current PCI–HN consists of 56 clinical items, which patients select from before their appointment, to help guide the outpatient consultation through the symptoms and problems that they may experience following their treatment for HNC. It helps focus consultations, aids doctor–patient communications, and assists in routing patients to other professionals for advice and support. Delphi research [11] was carried out to determine domains for it, with the 56 PCI items falling into 4 domains: (1) Physical and functional well-being (29 items), (2) treatment related (4 items), (3) social care and social-well-being (9 items) and (4) Psychological, emotional and spiritual well-being (14 items). Patients can also state any other items as a free-text response.

The PCI–HN approach is not a conventional screening process with cutoff and case-ness rather it provides opportunities for patients to raise issues they feel are important and that they want to discuss. Reduction techniques, to achieve optimal discriminatory properties with minimal input required from patients, are inappropriate. Content is what really matters with the PCI–HN and any item of concern selected by a patient is important. In selecting items some patients inevitably will under-estimate and others will over-estimate the gravity of what it is that concerns them. While it is unlikely that patients would select items for discussion that they did not want to discuss it is possible that some patients decline items that they feel they ought to discuss, because of embarrassment (e.g., alcohol, intimacy or financial problems), inappropriate environment or clinician. As far as is known no important aspect has been omitted, and there has been no obvious need to revise the PCI–HN since 2012. Content and face validity are thus relevant and all that is required is to understand the meaning of each item. The involvement of patient groups in the original design and updating of the PCI–HN argues in favour of such validity. The fact that the PCI–HN can be used as a screening tool [12] for adverse HRQOL does also indicate that it has desirable predictive properties.

### PCI–HN feasibility and acceptability

A survey of British Association of Head and Neck Oncology Nurses found that they preferred the PCI–HN with most (60%) feeling that, as a head and neck specific tool, the PCI–HN was most appropriate [13]. Research suggests (Table 1) the PCI–HN is appreciated by patients, they would like to continue using it in clinic and it is feasible to do so.

Pre-consultation PCI prompt lists can be completed electronically using touch screen technology (TST) or on paper. The prompt information is then available to the clinician and patient in real-time for use during the consultation. For our studies the PCI–HN was almost exclusively completed by electronic means. The TST approach is embedded into clinical practise at Aintree, with patients being approached in the waiting room by a hospital volunteer trained in administering the PCI, and it is very unusual for a patient to refuse. Most patients are willing and able to complete the PCI–HN on their own in the waiting room with others requiring the help of the volunteer in a designated room in the outpatient area. TST data is retrieved in real time by the clinician in another room in clinic immediately before the patient consultation. On the rare occasion of technology failure a paper version of the PCI can be completed and taken by the patient into the consultation. The first research paper about the PCI–HN [5] reported on a 28-week period from August 2007, involving 150 HNC patients of one consultant, three-quarters with oral cavity tumours, of whom all but 27 used the Touch-Screen Technology (TST). Only 3 of the 27 refused, the remainder being missed for various reasons at that time, either because there was a problem with the setting up of the PCI–HN at the start of clinic, or occasionally the system crashed for part of the clinic, or a few were taken to participate in another outcomes study and hence missed the PCI. The median (IQR) time for first completing the TST (PCI–HN/WQOL) was 8 (6–11) min with subsequent TST completions being shorter. The main reason for having difficulties with the TST was not having reading glasses, and as a result the clinic invitations now ask patients to bring their glasses. Although the PCI–HN did raise many issues it did not noticeably prolong the consultation (median 8 vs 7 min). Half of the PCI–HN patients felt it had made ‘quite a bit’ or ‘very much’ of a difference to their consultation. Typical comments were that it was ‘a bit more personal’, ‘reminds them of the points they want discussed’, ‘allows the consultation to get straight to the point’. Later research [14] with 454 clinics of the same consultant, gave an updated median (IQR) time for completing the TST as 8 (5–10) min, with these clinics being a median (IQR) of 18 (8–47) months after diagnosis of HNC.

A recent randomised trial of the PCI–HN involving 15 consultants from two separate units (Aintree & Leeds) and 288 HNC patients (47% oral cavity, 32% oropharynx, 14% larynx and 8% other) treated 2017–2019 [15] showed that the PCI–HN did not impact on the timetabling of clinic sessions. A 2013 [16] evaluation of the PCI–HN within the Merseyside and Cheshire Network recruited 66 patients, 8 doctors, and 6 nurse specialists, with patients being interviewed by telephone about 4 months after their first use of the PCI–HN. Almost all of them found completion of the PCI–HN to be easy or very easy, with no significant problems in running appointments. Two-thirds felt that most or all of their selected PCI–HN items were discussed with none feeling that the consultation had been made worse. Two-thirds felt that communication with the doctor was helped by the PCI–HN. Most wanted to continue with the inventory in future and most doctors and specialist nurses saw the potential for clinical practice. Comments received from the health professionals suggested that it seemed likely that the incorporation of the PCI–HN into practice at each clinic and locality would be achieved in different ways.

### How many items are selected and which items are selected most?

**Fig. 2:**
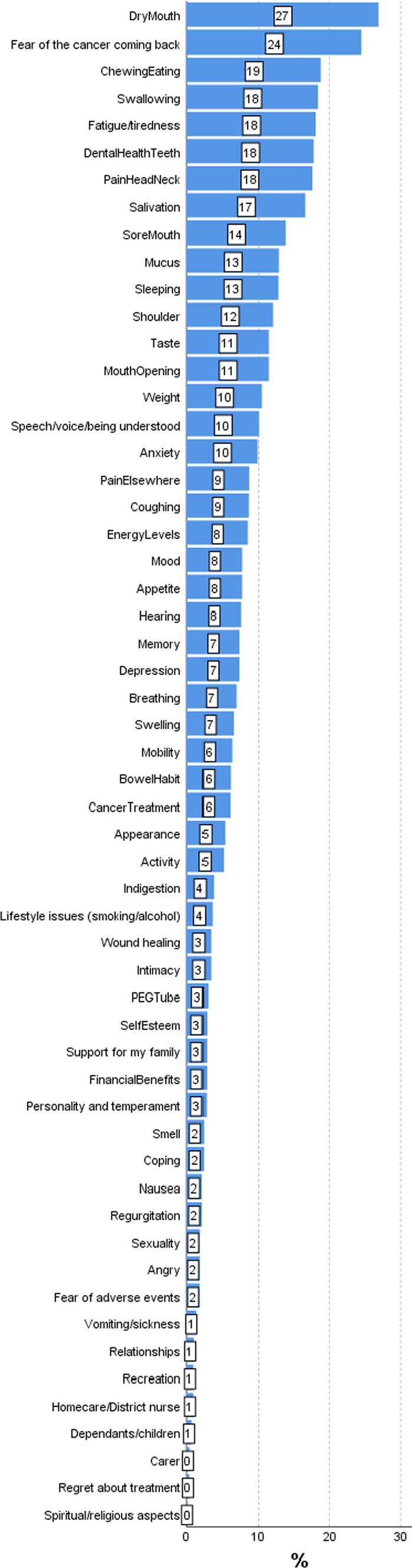
Frequency of items selected from the PCI record closest to 24 months after diagnosis, median (IQR) 23 (17–41) months

**Fig. 3:**
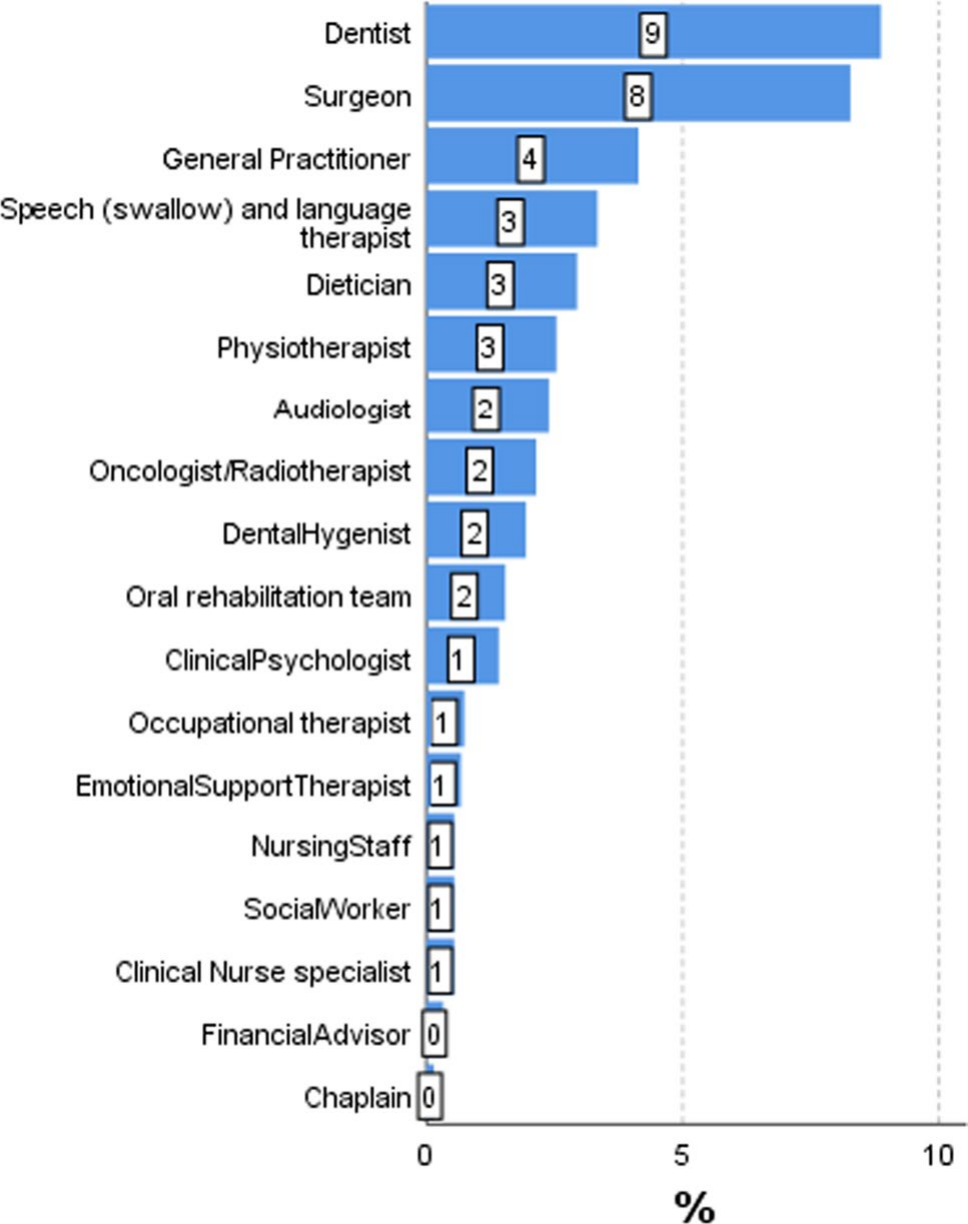
Frequency of allied professionals selected from the PCI record closest to 24 months after diagnosis, median (IQR) 23 (17–41) months

An accumulation of PCI–HN data for 507 patients were analysed specifically for this paper, with data from various studies ranging from the first patients seen in 2007 [5] to patients seen as part of the randomised trial up to 2020 [15]. The closest available PCI–HN record to 24 months after diagnosis was analysed, median (IQR) 23 (17–41) months. Median (IQR) age at diagnosis was 60 (54–69) years, 65% were male, tumour locations were oral cavity 52%, oropharynx 25%, larynx 13%, other HNC 11%. Early clinical stage (T1N0, T2N0) was 54%, advanced clinical stage 42%, stage unknown 4%. Treatment was surgery only 48%, surgery + adjuvant radiotherapy 36%, chemo/radiotherapy only 15%, treatment unknown 2%. Figure 2 shows the full range of PCI–HN items selected, the 10 main issues being of dry mouth (27%), Fear of cancer coming back (24%), chewing/eating (19%), swallowing (18%), Fatigue/tiredness (18%), dental health/teeth (18%), pain in head/neck (18%), salivation (17%), sore mouth (14%), mucus and sleeping (both 13%). The total number of items was a median (IQR) 2 (1–6) with 77% (392/507) selecting 1 or more items for discussion and mean 3.89. One or more items was selected by 70% (357) within the ‘physical and functional well-being’ domain (mean 2.77), 11% (55) within the ‘treatment related’ domain (mean 0.12), 18% (93) within the ‘social care and social-well-being’ domain (mean 0.24) and 40% (205) in the ‘Psychological, emotional and spiritual well-being’ domain (mean 0.78). Figure 3 shows the full range of professionals selected, with one or more professionals being selected by 28% (140), the most common being dentist (9%) and surgeon (8%).

Results from 140 PCI–HN patients within the PCI–HN trial [15] involving 15 consultants showed that with repeated use of the PCI–HN in routine follow-up clinics post treatment the number of items selected declines over time. At the trial baseline clinic, a median of 6.4 months after diagnosis, the median (IQR) number of items was 5 (2–9). At the final 12 month follow-up clinic for 100 PCI–HN patients, the median (IQR) number was 2 (0–4). At baseline the five most selected items were dry mouth (49%), dental health/teeth (34%), fear of cancer coming back (34%, chewing/eating (33%) and salivation (33%). At the final clinic the five most selected items were dry mouth (25%), salivation (18%), fear of cancer coming back (17%), pain in head/neck (17%) and fatigue/tiredness (17%). Nearly half, 46% (65/140) selected one or more allied professionals at baseline, most commonly dentist (16%), surgeon (14)%, oncologist/radiotherapist (9%), speech and language therapist (SLT) (8%) and dental hygienist (8%). After 12 months 18% (18/100) selected dentist (7%), surgeon (4)% and SLT (4%). The observed declined selection of items over time, could be due to a variety of reasons (may reflect adaptation, response shift), but may relate to the patient satisfaction after an intervention by the clinical team. Addressing an issue that cannot be resolved, rather than ignoring it, may also be a factor in the decline of the number of items selected.

In an international study involving 19 units from across the globe [17], with 2136 patients using a single sheet paper version of the PCI–HN, 27% of patients were within 12 months of diagnosis, 20% 12–23 months, 30% 24–59 months, and 23% ≥ 60 months. Contributing units reported little difficulty in translation of the PCI–HN and then in administering it. The median (IQR) total number of PCI–HN items selected by patients was 5 (2–10) with “fear of the cancer coming back” (39%) and “dry mouth” (37%) being common items amongst all units. Considerable variation was seen between units in the nature and number of items chosen, with some units selecting more items across all domains of the PCI–HN. This probably reflects differences in culture as well as patient expectations from previous use and capability of local healthcare systems. Unavailability of information about cancer treatment, superstition, illiteracy and differences in disclosure regarding doctor and patient communication, might also contribute. Cross-cultural differences will most probably exist in regard to family and care support and in the spiritual/existential aspects of living with cancer. A French study [18] of 72 HN Squamous cell carcinoma (SCC) patients who were alive and disease-free at least 1 year after treatment, mean 2.8 years, reported that the most frequently selected items were the fear of the cancer coming back (26%), dental health/teeth (25%), dry mouth (24%), fatigue (24%), speech/voice/ being understood (19%), chewing/eating (17%), and cancer treatment (15%). There were clear similarities between our studies in the UK and from international units regarding the most common items selected.

### Associations—with HRQOL and casemix

Data from 2007 to 2017 for one consultant (SNR) showed that patients at risk of specific adverse outcomes could be screened within appointments without any need for extra resources, staff or time [13]. Fifteen or more items selected on the PCI–HN was particularly predictive of emotional distress and of particular dysfunction across the social–emotional and physical domains of the UWQOL. Likewise, those selecting depression on the PCI–HN most probably have significant emotional distress. Such information can be used in real time during the consultation to help address unmet needs, to trigger for extra multidisciplinary assessment, and to timetable future appointments. The international study [17] involving 19 units showed a strong association between increased number of PCI–HN items and worse HRQOL. Female patients were more likely to select a greater number of items, especially those from the psychological, emotional and spiritual domain. Patients presenting with later stage tumours, patients having had radiotherapy and/or chemotherapy, and patients seen within 12 months after diagnosis were more likely to select a greater number of items. An increased number of PCI–HN items was associated with worse overall HRQOL. Binary regression was used to assess the association of each PCI–HN item with HRQOL being less than good and all 56 items had risk ratios over 1.00; it is, therefore, not so surprising that the total number of items selected emerged as such a strong indicator of overall HRQOL.

One paper [19] reported the use of the PCI across various HNC subsites (oral, oropharyngeal and laryngeal) and stages of disease (early and late). Consecutive Aintree patients treated for primary SSC HNC from 1998 to 2009 and alive in early 2011 were sent a postal survey containing the PCI–HN and UWQOL. They were asked to select PCI items to discuss with their consultant if the clinic was held that day. Survey response was 58% (447/775), with median follow-up of 3–4-year post-diagnosis across HNC subsites. The median (IQR) number of items selected varied from 2 (1–6) for early stage oral tumours to 6 (2–10) for late-stage oropharyngeal tumours and 7 (5–9) for late-stage laryngeal tumours. Fear of recurrence was common across tumour subsites (range 32–67%). Speech issues were more often raised by patients with laryngeal tumours, and saliva issues by those with oropharyngeal tumours. With the exception of early stage laryngeal tumours, patients consistently selected items concerning dental health/teeth and chewing. The median (IQR) number of allied professionals selected by patients was 1 (0–2), with little difference observed between HNC tumour subsites. Secondary analyses of the 140 PCI–HN patients from the PCI–HN trial [20] suggested that the number of PCI–HN items associated significantly with tumour stage and treatment though not with any of the measured deprivation indicators, such as the Index of Multiple Deprivation (IMD) at area level and financial benefits at patient level. The median (IQR) number of items for early stage tumours was 3 (2–6), while for advanced-stage tumours, it was 7 (3–11). For patients having surgery without radiotherapy/chemotherapy or free flap the median (IQR) number of items was 3 (1–5), otherwise it was 7 (4–10).

Using the accumulation of PCI–HN data for 507 patients at around 24 months after diagnosis specifically analysed for this paper, there were notable and significant differences (*p* ≤ 0.001) of treatment, tumour site and clinical stage with the number of items overall and the number of items in the Physical and functional well-being domain (Table 1). The strongest correlations with HRQOL were of the total number of items with the six-point overall QOL question (Spearman, rs = − 0.42) with the UW–QOL social–emotional subscale score (rs = − 0.55) and with the UW–QOL physical function subscale score (rs = − 0.50), all *p* < 0.001. Being elderly did not seem to affect the total numbers of PCI–HN items selected, though fewer were selected from the psychological, emotional and spiritual well-being domain, and in particular the item about fear of recurrence [21]. Dental health/teeth, chewing/eating, fatigue, swallowing and pain in H&N were consistently among the most commonly selected items regardless of age.

**Table 1:** Paper details and a brief summary of conclusions

Section	Paper	Conclusions
7	Aguilar et al. (2017) [47]	Dental concerns represent almost half of all PCI concerns observed in 10% or more of the sample patients.
4	Allen et al. (2016) [24]	With the PCI It is possible to identify the concerns of patients from lower socioeconomic strata as part of routine follow-up clinics. This allows for targeted multi-professional intervention and supports to improve the outcome in this hard to reach group.
7	Aminnudin et al. (2020) [48]	Routine follow-up consultations should incorporate the PCI-H&N prompt list to enhance patient-centred care approach as the type and number of patients’ concerns are shown to reflect their HRQoL and psychological distress
7	Breeze et al. (2016) [49]	Use of the Patient Concerns Inventory enables tailoring of services towards those clinicians who a patient feels are potentially most likely to help alleviate factors that have an adverse effect on QoL.
7	Broderick et al. (2020) [50]	Salivation response was associated strongly with the other measures of health-related quality of life (HRQoL) and the PCI.
5	Chieng CY et al. (2021) [35]	The Patient Concerns Inventory (PCI) and the University of Washington quality of life questionnaire (UW-QOL v4) were used. Pain was a major dysfunction (63%) as was physical and social-emotional functioning and this group reported many PCI issues, median (IQR) 7 (4–11)
7	Dimelow et al. (2021) [51]	A prompt list sent out to the patient in advance of the consultation (Patient Concerns Inventory) could be a useful adjunct in telephone consultations
3, 4	Elaldi et al. (2020) [18]	Identification of patient needs and concerns along with multidisciplinary management of persistent symptoms and psychological distress seem essential steps towards improving QoL of HNSCC patients.
6	Ezeofor et al. (2022) [44]	The PCI appears to be a low-cost intervention that generates a cost-effective benefit to patients from a UK National Health Service (NHS) perspective if rolled out as part of routine care. Qualitative evidence has shown that the use of the PCI is supported by consultants in routine practice.
5	Flexen et al. (2012) [28]	Patients who choose concerns about appearance for discussion on the UW-QoL questionnaire and not on the PCI risk being missed if only the PCI is completed. Both tools compliment the screening of patients who have problems with facial disfigurement; failure to identify them can have serious clinical and psychosocial implications.
5	Ghazali et al. (2011) [33]	Routine use of PCI promotes target efficiency by directing and apportioning appropriate services to meet the needs for supportive care of head and neck cancer survivors.
5	Ghazali et al. (2012) [26]	The use of both surveys concurrently enabled all patients with swallowing or speech issues to discuss these concerns in the clinic and to access appropriate multidisciplinary interventions.
7	Ghazali et al. (2012) [52]	The results of this study suggest that the UW-QOL with the worse-same-better modification should be used together with the PCI to allow optimal identification of issues for patient-clinician discussion during routine outpatient clinics.
5	Ghazali et al. (2013) [32]	This study confirmed that self-reported screening fear of recurrence (FoR) using the PCI is a valuable tool in identifying significant FoR.
5	Ghazali et al. (2013) [36]	The PCI also gives patients an opportunity to identify from a list of 15 multidisciplinary team (MDT) members whom they would like to see or be referred on to.
5	Ghazali et al. (2013) [37]	This approach gave an insight into the way the PCI mediates consultations, and how patients do not always understand the support that specific professionals can provide. Overall, patients were satisfied with the consultations.
1	Ghazali et al. (2015) [11]	The items selected under the HNC-PCI domains showed that certain clinical, pathological, and HRQoL factors were associated with specific patterns of needs or concerns.
4	Ghazali et al. (2017) [23]	A cutoff score ≥4 or ≥5 PCI items selected can identify those at risk of significant distress. Concerns causing significant distress were related to emotional/psychological issues and physical function.
7	Hatta et al. (2014) [53]	The PCI was considered feasible, thus favouring its future use in routine oral cancer patient management
5	Jungerman et al. (2017) [39]	The translation and cultural adaptation of the PCI into Brazilian Portuguese language was successful, and the results demonstrate its feasibility and usefulness, making this a valuable tool for use among the Brazilian head and neck cancer population.
2, 5	Kanatas et al. (2012) [14]	The results showed that the combination of the UW-QOL questionnaire and the PCI provide a practical means of screening for psychological distress in clinics.
4	Kanatas et al. (2013) [19]	Completion of the PCI by patients before consultation can highlight problems and concerns that doctors can target for discussion, thereby streamlining consultations, and ensuring that patient needs are better met, thus creating a more effective service.
5	Kanatas et al. (2015) [31]	Fear of recurrence is common but patients with multiple emotional concerns need additional support, and further research is required to specify the practical details of the interventions needed at various points during and after treatment.
7	Kanatas et al. (2020) [46]	Our preliminary experience is that the HaNC-AD PCI may provide a very useful tool prior to treatment delivery during this crisis, with information delivered remotely by the clinical team.
7	Kanatas et al. (2020) [54]	Donor site morbidity, in our patient sample, did not appear to be an issue that patients wanted to discuss.
7	Kanatas et al. (2020) [55]	The preliminary experience is that the PCI-HN may provide a very useful tool to aid remote consultations, but more clinical evidence is needed in order to ensure that such consultations are optimal for our head and neck patients.
5	Mahmood et al. (2014) [27]	Better communication with GDPs is essential.
1	Miller et al. (2018) [10]	The PCI-HN is specific for HNC and differs from many QPLs, which are more general cancer tools.
6	Mortensen et al. (2022) [62]	Nursing rehabilitation consultations using the Patient Concerns Inventory are feasible and may ensure that patient preferences and priorities are incorporated in their care.
7	Ozakinci et al. (2018) [56]	Analyses indicate that patients may feel reluctant to raise their fear of cancer recurrence with their clinician for fear of appearing “ungrateful” or of damaging a relationship that is held in high esteem.
1, 2, 3	Rogers et al. (2009) [5]	The Patients Concerns Inventory (PCI) helps focus the consultation onto patient needs and promotes multidisciplinary care. Following this very successful pilot the PCI is being rolled out to other consultants in the H & N clinic.
7	Rogers at al (2018) [58]	The inventory allows for greater opportunity to provide holistic targeted multiprofessional intervention and support.
7	Rogers et al. ( 2010) [57]	FOR is a common concern and because it is not possible to identify patients based on clinical parameters, it is important to screen for FOR to direct patients to appropriate support and intervention.
5	Rogers et al. (2012) [29]	The UW-QOL and PCI package is a valuable tool that may routinely screen for significant pain in outpatient clinics.
2	Rogers et al. (2014) [16]	It is likely that the PCI-HN will be incorporated into practice at each clinic and locality in different ways.
4	Rogers et al. (2015) [21]	It is possible to recognise concerns in routine clinical care, thus allowing the opportunity for intervention and support to improve the outcome for the elderly.
5	Rogers et al. (2015) [30]	The PCI identified that 9 of the 24 reporting the worst problems wanted the topic discussed in clinic, and clinic letters suggested that 5 of these discussed the issue in clinic with 4 being referred on, 3 to a clinical psychologist and 1 to a clinical nurse specialist.
4	Rogers et al. (2016) [22]	The total number of PCI items selected is a useful predictor of QoL.
5	Rogers et al. (2017) [40]	This study helps to inform resource allocation (assistance and clinic area) when adopt the PCI across the whole oncology outpatient setting. Further research is needed to identify cost efficient ways to promote the self- completion of the PCI in those patients less confident
5, 7	Rogers et al. (2018) [34]	The diversity of concerns raised by patients highlights the need for holistic assessment during follow up, and integration of the inventory into routine consultations will mean that we can repeat it.
6	Rogers et al. (2018) [41]	This trial will provide knowledge on the effectiveness of a consultation intervention package based around the PCI used at routine follow-up clinics following treatment of head and neck cancer with curative intent
3, 6, 7	Rogers et al. (2019) [17]	There was considerable variation between units in the number of items selected and in overall QOL, even after allowing for case-mix variables. There was a strong progressive association between the number of PCI items and QOL.
2, 3, 6	Rogers et al. (2020) [15]	This novel trial supports the integration of the PCI approach into routine consultations as a simple low-cost means of benefiting HNC patients.
4	Rogers et al. (2020) [25]	PCI fatigue was common in those with sleeping, nausea, mood, depression, mobility, breathing, and energy level concerns.
6	Rogers et al. (2020) [43]	The inclusion of a prompt list to help facilitate conversation with patients did not make a substantial difference to consultation times.
4	Rogers et al. (2021) [20]	Interventions designed to improve employment and finance could make a substantial positive effect on HRQOL outcomes and concerns
6	Rogers et al. (2021) [61]	Around one in ten HNC patients attending routine outpatient follow-up consultations report high fear of cancer recurrence (FCR), however for female patients under the age of 55 the rate was one in three.
7	Rushworth et al. (2018) [45]	Whilst there has been extensive research into the use of the post treatment PCI, there is little information on the benefits of the use of PCI (at diagnosis). Further research is required in order to establish its role and timing in the cancer journey. This may have important implication in patient care.
5	Scott et al. (2013) [38]	This study found that the paper version of the PCI was an acceptable alternative to the touch-screen technology version.
7	Semple et al. 2018) [59]	Providing a patient-focused follow-up consultation can facilitate the identification of unmet needs, permitting timely and appropriate intervention being initiated.
1	Shunmugasundaram et al. (2019) [4]	The PCI can be used to measure unmet needs in the HNC setting considering the importance of content validity over quantitative psychometric properties.
7	Shunmugasundaram et al. (2021) [60]	The translated Hindi Patient concerns inventory is conceptually and linguistically validated and equivalent with the original English version.
1,5	Twigg et al. (2021) [12]	The single-sheet prompt list enables clinicians to identify patients at high risk of poor HRQOL. This PCI-HN approach has the potential to be integrated into routine clinical practice.
1	Wells et al. (2015) [13]	The diversity of concerns and unmet needs identified in this study highlights the importance of holistic needs assessment as part of follow-up care for HNC survivors with tailoring of support for particular concerns.

Sections:

1. What is the PCI-HN and how does it work?

2. PCI-HN feasibility and acceptability

3. How many items are selected and which items are selected most?

4. Associations—with HRQOL and casemix

5. Other observational studies

6. Randomised trial of the repeated use of the PCI-HN

7. General* Discussion and further research*

Clinical characteristics can predict some problems, notably oral function, whereas the PCI–HN is a more sensitive indicator of overall QoL, particularly the total number of PCI–HN items selected by patients [22]. The total number is simple to compute, and is associated mainly with overall QoL, though we did find clinically relevant gradients in physical and social–emotional subscale scores. In one study involving 4 participating consultants and 170 prospectively recruited patients without any previous experience of the PCI–HN, the number of PCI–HN items selected was a possible proxy marker of significant distress at 2 years after diagnosis as measured by the distress thermometer [23]. Experiencing significant distress and raising numerous PCI–HN concerns also impacted upon the length of the consultation.

For consecutively diagnosed patients 2008–2012, median (IQR) months to first clinic 4 (2–10) [24], there were no notable differences seen in respect of IMD classification by the number and type of PCI–HN items selected at their first PCI–HN clinic. It might have been expected that patients living in lower socio-economic status (SES) and deprived neighbourhoods would choose fewer items to discuss in their consultation. PCI–HN trial baseline data identified ‘fatigue/tiredness’ following treatment for head and neck cancer to be the sixth most commonly selected [25], by 29% (*n* = 40/140). Patients with advanced tumours were significantly more likely to have selected the fatigue item (36% vs 18%), as were those patients treated with radiotherapy/chemotherapy (39% vs 11%). Patients selecting PCI–HN fatigue/tiredness reported significantly worse overall quality of life, social–emotional and physical function composite scores (UWQOL), as well as worse Distress Thermometer scores and European Quality-of-Life Five Dimension Five level (EQ-5D-5L) scores. PCI fatigue was more often observed in those patients with sleeping, nausea, mood, depression, mobility, breathing, and energy level concerns.

Elaldi et al. [18], used a French translation of both the PCI–HN prompt list and of the European Organisation for Research and Treatment of Cancer (EORTC) HRQOL questionnaire. They reported a negative correlation between the total number of patient concerns selected and the mean score for functioning scales (*r* =  − 0.43) and a positive correlation with the mean scores for general (*r* = 0.49) and for head and neck symptoms (*r* = 0.45). A similar tendency, not statistically significant, was seen for correlations between QoL scores and the number of staff members selected by patients. Gender (*p* = 0.002) was associated with the number of patient concerns, and patient age (*p* = 0.003) with the number of staff members selected.

### Other observational studies

Numerous research papers have reported on the accumulating data set arising from the use of the PCI–HN and UW–QOLv4 by the same consultant (SNR) at Aintree since 2007, each paper with a separate distinct focus. Several papers investigated the concurrent use of the PCI–HN and UW–QOL as a means of identifying concerns and case-ness for mood and anxiety [14], swallowing and speech [26], chewing and dental issues [27], appearance [28], pain [29], intimacy and sexuality [30], the full range of emotional concerns [31] and longitudinal trends in Fears of Recurrence [32]. Results showed that the use of both UW–QOL and PCI–HN in clinics offers a practical means of screening for psychological distress [15]. The PCI–HN can identify previously undetected swallowing and/or speech dysfunction as well as significant swallowing or speech issues in those not wanting to discuss them, perhaps because of acceptance and adaption to their deficits [26]. Patients with significant chewing problems should be encouraged to obtain a dentist and this should be a priority to improve shared care and perhaps alleviate their chewing problems [27]. Surgical treatment for patients with more advanced disease predisposes them to having more visible disfigurement in their appearance, with greater psychological costs, and more negative effects on daily living and overall quality of life [28]. Patients self-reporting significant pain or patients wishing to discuss pain had problems more often in physical and social–emotional functioning, with sub-optimal overall QOL and they raised more non-pain PCI–HN items for discussion, including depression and anxiety [29].

The notion that routine screening for unmet needs prompts larger numbers of referrals and greater burden on healthcare services is unfounded [33]. The first study to assess the frequency of completion of the PCI–HN involved a consecutive sample of 92 patients treated curatively for oral cancer between January 2008 and December 2011, with all clinic attendances reviewed until 2015 [34]. The completion rates were disappointing, reflecting approach issues. Although very few patients actually refused the PCI–HN, the rates reflect the reality of a busy clinic, and it was not a rigorous clinical trial. The iPadTM system was available only in the afternoon of the all-day oncology clinic, and this relied on a volunteer being present, which explains much of the missed opportunity. A review of the availability and use of the PCI–HN over its first 7 years indicated 386 patients completed 1198 inventories at 220 clinics, median 6 (range 4–7) per clinic, where median time to first clinic was 10 months after diagnosis. Apart from technical issues at the beginning of 2012, the use of the inventory was maintained at similar levels (median six per clinic to October 2011 and median five per clinic from July 2012).

A study of ORN patients with follow-up clinic data 2008–2020 indicated that HNC patients with ORN progression reported an average of nine issues [35], double the average seen in typical HNC follow-up consultations. One study evaluated the introduction of the PCI–HN into clinical practice, where both doctor and patients were unfamiliar with it [36]. Cancer treatment’ was the most discussed issue in 60–70% of consultations, regardless of whether or not the PCI–HN was used and the largest amount of consultation time went on biomedical issues. While both the number and the range of items of concern discussed during consultations increased with using the PCI, the length of consultation remained relatively unchanged. In a study to produce a thematic framework for rating items discussed in a PCI–HN-mediated consultation [37], the two assessors agreed for 80% (65/81) of consultation audio recordings. The median number of items selected on the PCI–HN before the consultation was 4, compared to 6 actually discussed during the consultation. Regarding professional involvement, the medians were 0 and 3, respectively. Some PCI–HN items that are rarely selected, such as relationships, were often discussed during consultations, while others chosen on the PCI–HN were sometimes not discussed largely because of insufficient time in the consultation to discuss all items selected. The PCI–HN could, therefore, be used to select patients requiring more time, and clinical nurse specialists could provide this contact after the consultation with the doctor has ended. Another study compared paper and touch-screen technology (TST) versions of the PCI–HN, involving 2 consultants [38] and the paper version was found to be an acceptable alternative. No significant differences between paper and TST were found in the number of PCI–HN items selected beforehand, items discussed in consultation, or in the length of consultation.

One study successfully translated the PCI–HN into the Brazilian Portuguese language, culturally validated it and evaluated in a consecutive series of Brazilian patients [39].In another study to evaluate how easy and confident patients felt about using an iPad to complete the PCI–HN without assistance in a busy oncology outpatient clinic setting [40] the practical implication was that three-quarters of patients were willing and able to complete the iPad in the waiting area without involving the volunteer. Lack of confidence in this was largely a generational influence with older patients being less computer savvy or having problems with their eyesight (or forgetting their reading glasses).

### Randomised trial of the repeated use of the PCI–HN

Although the PCI–HN had been well received by patients and was being adapted for use with other conditions, a trial was needed to demonstrate its efficacy because of the power and robustness level 1 evidence gives to inform, shape and transform clinical practice and patient care. The main aim of the trial was to explore whether the routine use of the PCI–HN in review clinics during the first year following head and neck cancer treatment could improve patients’ quality of life [15, 41, 42]. Consultation times were unaffected [43] and the number of PCI–HN items selected decreased over time. Primary pre-stated analyses indicated a small statistically significant clinical effect of PCI–HN intervention on UW–QOL social–emotional subscale scores, while overall quality of life results favoured the PCI–HN group without achieving statistical significance. Secondary exploratory analyses indicated that HRQOL status early after the completion of treatment was the dominant predictor of HRQOL after another 12 months and the trend in analyses over a range of outcomes suggests that patients with worse early HRQOL could benefit more from the PCI–HN. The PCI–HN is a low-cost intervention which generates a cost-effective benefit to patients from an NHS perspective if rolled-out as part of routine care [44]. The trial findings indicated that a pragmatic multi-unit cluster trial for a study of this nature was feasible, and it has helped increase the profile of this type of approach in clinical care as well as increasing the body of evidence with publications in peer review journals.

### General discussion and further research

Research so far on using the PCI–HN strongly suggests that it has a positive impact on outcome. Patients definitely perceive the benefit and appreciate the approach especially when having a consultation with someone unfamiliar or less experienced. The PCI approach is a driver for improvement in patient–clinician communication in routine practice. The prompt list has increased awareness amongst clinicians of the needs of patients post-treatment, with a keen focus on improving the patient experience and the understanding of person-centred practice.

Limitations of this work include that many of the papers analysed data from just two UK centres (Liverpool and Leeds) and is possible that these results may not apply worldwide, though one study did involve patients from 19 units in 16 countries. To globalise the benefits of the PCI–HN one of the most important steps is the cultural validation [39], as some words and expressions may be mis-understood in different parts of the world. Additional items may not be required, but the translation process needs to consider cultural adaptation and different healthcare settings. The wider use of prompt lists has implications for improving patient–doctor communication and education in this area. The research serves to underpin the tools used to empower patients to identify unmet needs, such as the eHolistic Needs Assessment/Distress thermometer. With the trial complete by far the main focus now has to be the roll-out the PCI–HN and how best to bring this about. Macmillan’s cancer specific eHNA resource has used PCI–HN items to create a head and neck specific eHNA extended module. It is possible that the prompt list approach in other cancers will support the development of additional cancer type specific modules. There is also a PCI–HN variant suitable for use with patients at the time of diagnosis that has been used effectively in telephone consultations [45, 46].

Various research and clinical teams have contributed with papers (Table 2) relating to the PCI–HN and this is a further endorsement of its benefit. Wider uptake of the PCI–HN beyond the consultant clinic could be promoted on the available evidence. Further research could focus on other professionals who conduct follow-up clinics in routine practice, such as Clinical Nurse Specialists and Speech and Language Therapists, as well as less experienced medical trainees. There is also scope to develop and evaluate other PCI modules in cancer and chronic conditions in primary care. It would also be interesting to explore the use of a PCI for carers. Furthermore, research concentrating on how the PCI is working in the consultation is a critical step to providing a robust understanding of its mechanism of action. This understanding will inform future initiatives aimed at improving the use and efficacy of the PCI–HN.

**Table 2:** Associations and casemix

			Total	D1	D2	D3	D4	STAFF
	Total	507	2 (1–6) 3.89	2 (0–4) 2.77	0 (0–0) 0.12	0 (0–0) 0.24	0 (0–1) 0.78	0 (0–0) 0.41
Gender	Male	327	2 (1–6) 4.03	2 (0–4) 2.92	0 (0–0) 0.13	0 (0–0) 0.25	0 (0–1) 0.74	0 (0–1) 0.48
	Female	180	2 (0–6) 3.63	1 (0–4) 2.49	0 (0–0) 0.09	0 (0–0) 0.22	0 (0–1) 0.84	0 (0–0) 0.27
		*p* value	0.40	0.10	0.18	0.94	0.18	0.02
Age at diagnosis	<55	142	2 (0–6) 3.66	1 (0–3) 2.39	0 (0–0) 0.06	0 (0–0) 0.25	0 (0–1) 0.96	0 (0–1) 0.38
	55–64	197	2 (1–7) 4.47	2 (0–5), 3.14	0 (0–0) 0.19	0 (0–0) 0.28	0 (0–1) 0.87	0 (0–1) 0.45
	65–74	114	3 (1–5) 3.62	2 (1–3), 2.76	0 (0–0) 0.10	0 (0–0) 0.22	0 (0–1) 0.54	0 (0–1) 0.37
	75+	54	2 (1–4) 2.94	2 (0–4) 2.43	0 (0–0) 0.06	0 (0–0) 0.07	0 (0–1) 0.41	0 (0–1) 0.39
		*p* value	0.16	0.52	0.005	0.16	0.24	0.96
Treatment	Surgery only	241	2 (0–5), 3.21	1 (0–3) 2.09	0 (0–0) 0.07	0 (0–0) 0.23	0 (0–1) 0.83	0 (0–1) 0.38
	Surgery + adjuvant RT	180	3 (1–6), 4.22	2 (1–5) 3.16	0 (0–0) 0.13	0 (0–0) 0.23	0 (0–1) 0.71	0 (0–1) 0.39
	Chemoradiotherapy only	77	3 (1–8), 5.16	2 (0–7) 3.94	0 (0–0) 0.21	0 (0–0) 0.29	0 (0–1) 0.74	0 (0–1) 0.52
		*p* value	0.001	<0.001	.003	.99	.86	085
Tumour site	Oral cavity	261	2 (1–5) 3.37	1 (0–3) 2.31	0 (0–0) 0.08	0 (0–0) 0.25	0 (0–1) 0.74	0 (0–1) 0.39
	Oropharynx	125	5 (1–8) 5.58	3 (1–7) 4.13	0 (0–0) 0.18	0 (0–0) 0.30	0 (0–1) 0.98	0 (0–1) 0.53
	Larynx	64	2 (0–3) 2.80	1 (0–3) 2.13	0 (0–0) 0.13	0 (0–0) 0.14	0 (0–1) 0.41	0 (0–1) 0.39
	Other	57	2 (1–6) 3.81	2 (0–5) 2.61	0 (0–0) 0.11	0 (0–0) 0.18	0 (0–1) 0.93	0 (0–0) 0.23
		*p* value	<0.001	<0.001	0.16	0.41	0.03	0.24
Clinical stage	Early 1–2	272	2 (0–5) 3.21	1 (0–3) 2.17	0 (0–0) 0.10	0 (0–0) 0.22	0 (0–1) 0.74	0 (0–1) 0.40
	Advanced 3–4	213	3 (1–8) 4.85	2 (1–6) 3.62	0 (0–0) 0.15	0 (0–0) 0.26	0 (0–1) 0.83	0 (0–1) 0.42
		*p* value	<0.001	<0.001	0.04	0.69	0.33	0.87
Months from diagnosis To clinic (quartiles)	9.00–16.53	127	2 (0–6) 4.31	2 (0–4) 3.14	0 (0–0) 0.14	0 (0–0) 0.25	0 (0–1) 0.79	0 (0–1) 0.44
	16.54–23.23	126	2 (1–5) 3.77	2 (0–4) 2.79	0 (0–0) 0.13	0 (0–0) 0.20	0 (0–1) 0.67	0 (0–1) 0.46
	23.24–40.97	128	2 (0–6) 3.84	2 (0–4) 2.84	0 (0–0) 0.08	0 (0–0) 0.20	0 (0–1) 0.73	0 (0–1) 0.33
	≥40.98	126	2 (1–6) 3.63	2 (0–3) 2.30	0 (0–0) 0.12	0 (0–1) 0.30	0 (0–1) 0.91	0 (0–1) 0.40
		*p* value	0.97	0.66	0.37	0.06	0.48	0.75

*p* value: Mann–Whitney test (two group comparison) or Kruskal–Wallis test (three or more group comparison)
